# Identification and validation of an anoikis-related genes signature for prognostic implication in papillary thyroid cancer

**DOI:** 10.18632/aging.205766

**Published:** 2024-04-24

**Authors:** Runyu Zhao, Yingying Lu, Zhihan Wan, Peipei Qiao, Liyun Yang, Yi Zhang, Shuixian Huang, Xiaoping Chen

**Affiliations:** 1 Postgraduate Training Base at Shanghai Gongli Hospital, Ningxia Medical University, Shanghai 200135, China; 2 School of Medicine, Shanghai University, Shanghai 200444, China; 3 Department of Endocrinology, Gongli Hospital of Shanghai Pudong New Area, Shanghai 200135, China; 4 Department of Otolaryngology Head and Neck Surgery, Gongli Hospital of Shanghai Pudong New Area, Shanghai 200135, China

**Keywords:** papillary thyroid cancer, anoikis, tumor microenvironment, prognostic model, nomogram

## Abstract

Thyroid cancer, notably papillary thyroid cancer (PTC), is a global health concern with increasing incidence. Anoikis, a regulator of programmed cell death, is pivotal in normal physiology and, when dysregulated, can drive cancer progression and metastasis. This study explored the impact of anoikis on PTC prognosis. Analyzing data from GEO, TCGA, and GeneCards, we identified a prognostic signature consisting of six anoikis-related genes (ARGs): EZH2, PRKCQ, CD36, INHBB, TDGF1, and MMP9. This signature independently predicted patient outcomes, with high-risk scores associated with worse prognoses. A robust predictive ability was confirmed via ROC analysis, and a nomogram achieved a C-index of 0.712. Differences in immune infiltration levels were observed between high- and low-risk groups. Importantly, the high-risk group displayed reduced drug sensitivity and poor responses to immunotherapy. This research provides insights into anoikis in PTC, offering a novel ARG signature for predicting patient prognosis and guiding personalized treatment strategies.

## INTRODUCTION

Thyroid cancer (THCA) is the most prevalent endocrine malignancy worldwide, with its incidence and morbidity steadily increasing [1]. Papillary thyroid cancer (PTC) is the predominant subtype of THCA, accounting for approximately 80% of all THCA cases [2]. The prognosis for PTC is typically favorable, with a 5-year survival rate exceeding 95% and a 10-year survival rate over 90% when treated with radioiodine ablation and/or revision surgery [3]. But it is important to note that approximately 20% of patients experience a reduced survival rate due to factors such as recurrence, metastasis, and other complications [4, 5]. Therefore, exploring the key molecules that promote the progression of PTC is necessary to improve the prognosis and quality of life of PTC patients [6].

Anoikis is a term used to describe a form of programmed cell death triggered by the detachment of cells from the extracellular matrix (ECM) or neighboring cells [7]. It is a crucial mechanism that regulates tissue homeostasis, development, and the prevention of cancer cell metastasis. When cells lose contact with their surrounding ECM, they undergo a series of biochemical and morphological changes that ultimately lead to their death [7]. During anoikis, various signaling pathways are activated, including integrin-mediated signaling, growth factor receptor signaling, and apoptotic pathways. These pathways converge to induce apoptosis, characterized by DNA fragmentation, mitochondrial dysfunction, cytoskeletal rearrangement, and caspase activation [7, 8]. Anoikis plays a significant role in normal physiological processes such as tissue remodeling and the maintenance of epithelial cell layers [9, 10]. Dysregulation of anoikis can have detrimental effects, contributing to cancer progression and metastasis [8]. Cancer cells often acquire resistance to anoikis, allowing them to survive and invade distant tissues [11].

Recently, there is a growing number of research focusing on the significance of anoikis resistance in tumorigenesis. The endocytic degradation of epidermal growth factor receptor (EGFR) can induce cancer cells to detach from the extracellular matrix, ultimately triggering apoptosis, known as anoikis. Targeting EGFR could be effective for anoikis suppression [12]. Upon stimulation by specific extracellular matrix components, such as cancer-related fibroblasts (CAFs), tumors acquire anoikis resistance and develop invasive and metastatic capabilities [13]. Emerging studies have demonstrated that the anoikis-related signature holds the potential to predict treatment response and prognosis in a wide range of cancer types. Until now, the establishment of clinical prognostic models based on anoikis-related genes has explored in head and neck squamous cell carcinoma, clear cell renal cell carcinoma, hepatocellular carcinoma, and others [14–16], but remains unexplored in PTC. Gaining insight into the mechanisms of action of anoikis-related genes may enhance our understanding of the development process of PTC.

In this study, we conducted an analysis of differentially expressed genes (DEGs) between PTC and adjacent normal tissues using publicly available databases. By integrating this data with clinical information, we developed a six-gene signature related to anoikis to predict the prognosis of PTC patients and elucidate immune cell infiltration patterns. This approach aims to enhance treatment options and improve patient outcomes in PTC.

## MATERIALS AND METHODS

### Data acquisition

The messenger RNA (mRNA) expression data of PTC and normal tissues were downloaded from the GEO (including the GSE29265, GSE33630, and GSE60542 datasets; https://www.ncbi.nlm.nih.gov/geo). Additionally, the mRNA sequencing data (FPKM value) and corresponding clinical-pathologic information of PTC patients were obtained from the TCGA (https://cancergenome.nih.gov/). A total of 498 PTC patients were obtained and randomly assigned to either the training cohort (*n* = 349) or the test cohort (*n* = 149).

### Identification of anoikis-related DEGs and functional enrichment analysis

Anoikis-related genes (ARGs) were extracted from GeneCards [17]. In the GeneCards database, each gene has a corresponding relevance score value, which is used to evaluate the correlation between the gene and the elements (chemical substances and diseases). The higher the score, the stronger the statistical correlation between genes and related elements [17]. Using a relevance score >0.4, 551 ARGs were selected. The “limma” R package was employed to screen DEGs (|log2(fold change)| > 1 and adjusted *P*-value < 0.05) between tumor tissue and normal tissues in GEO datasets. Then, the anoikis-related DEGs (ADGs) were obtained by overlapping the intersection of DEGs and ARGs using Venny (version 2.1) [18]. The ADGs were submitted to Metascape Online (https://metascape.org/), which incorporates a core set of default ontologies such as Gene Ontology (GO) and Kyoto Encyclopedia of Genes and Genomes (KEGG) pathways to conduct functional analysis and construct a PPI network [19].

### Establishment of the prognostic genes signature of the ADGs

To establish a prognostic model for ADGs in PTC patients from TCGA database, a univariate Cox analysis of progression-free interval (PFI) was first conducted using the R packages “survival” and “survminer” to identify the survival-related genes among the ADGs with a prognosis value (*P* < 0.05). These survival-related genes were then included in the subsequent Least Absolute Shrinkage and Selection Operator (LASSO) Cox regression analysis by using “glmnet” R package. The 10-fold cross validation and 1,000 iterations were performed to minimize the potential risk of overfitting and select optimal prognostic genes. The prognostic genes signature was calculated based on the normalized gene expression level and corresponding regression coefficients. The risk score for prognosis was determined by using a linear combination of the regression coefficient in the LASSO regression and the gene’s expression level.

### The assessment of the prognosis model

PTC patients in both the training cohort and test cohort were respectively divided into high- and low-risk groups by the risk scores. The Kaplan-Meier (K-M) survival curve was performed to assess prognostic significance. The receiver operating characteristic curve (ROC curve) was created by using the R package “timeROC.” The area under the curve (AUC) in 1-, 3-, and 5-year progress-free survival was used to evaluate the performance of the prognostic model. The GEPIA database (http://gepia.cancer-pku.cn/) was used to perform survival analysis for the prognostic genes [20].

### Nomogram construction and evaluation

Univariate and multivariate Cox analyses of patient risk score of the prognostic signature and clinical factors, including gender, pathological stage, and focal type, were performed to determine the significance of each factor in predicting PFI in patients with PTC. Based on the results of multivariate Cox regression, the R package “rms” was used to construct a nomogram to provide the 1-, 3- and 5-year survival probabilities. The calibration curve evaluated the predictive ability of the nomogram.

### Functional analysis related to risk score

The “limma” R package was employed to screen DEGs (|log2(fold change)| > 1 and adjusted *P*-value < 0.05) between high- and low-risk groups in TCGA database. Heatmap was performed using the “pheatmap” and “ggplot2” R packages. GO and KEGG enrichment analyses were utilized to explore the biological functions of DEGs.

### Evaluation of tumor immune microenvironment

The ESTIMATE algorithm was used to calculate the stromal score, immune score, and tumor purity between high-risk and low-risk groups [21]. The CIBERSORT algorithm was used to estimate the abundance of 22 immune cell types. The infiltration levels of 22 immune cell types between the high-risk and low-risk subgroups were compared [22].

### Drug sensitivity and immunotherapeutic response analyses

The “pRRophetic” R package was employed to calculate the half-maximal inhibitory concentration (IC_50_) values of chemotherapeutic and targeted drugs for each PTC patients in TCGA cohort [23]. The tumor immune dysfunction and exclusion (TIDE; http://tide.dfci.harvard.edu/) was used to calculate the TIDE score of each patient according to myeloid-derived suppressor cell (MDSC), macrophage M2, T cell Dysfunction and Exclusion [24].

### Statistical analysis

R software (version 4.3.0) was used to perform data analysis. The statistical value *P* < 0.05 indicates that the difference is statistically significant.

### Data accessibility

The datasets used and/or analyzed during the current study are available from the corresponding author on reasonable request.

## RESULTS

**Figure 1:**
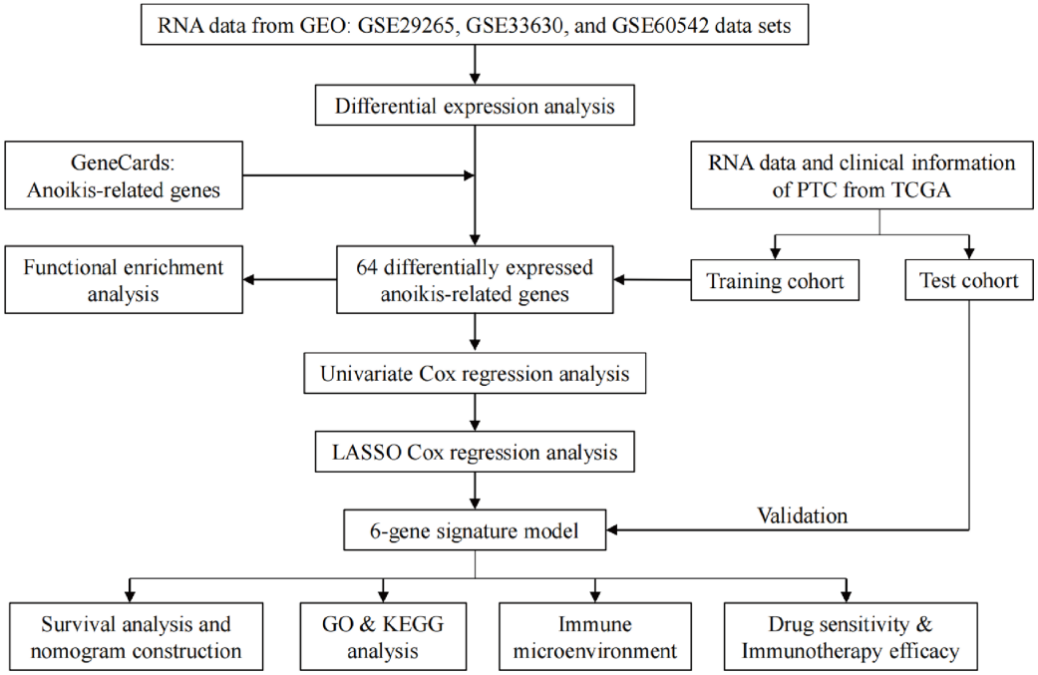
Flow chart of the current study.

Figure 1 illustrates the cohort design and analytical concepts for the entire study.

### ADGs in PTC patients and functional enrichment analysis

**Figure 2:**
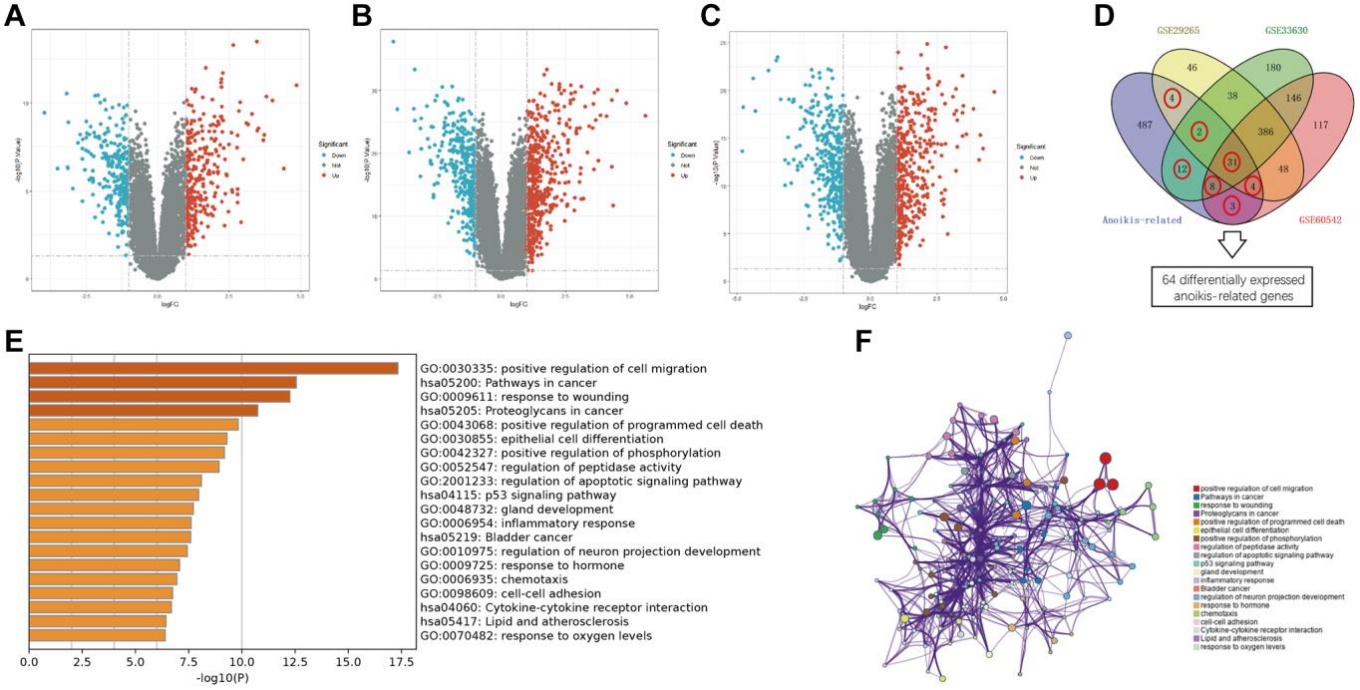
**Overview of the differentially expressed anoikis-related genes in PTC.** (**A**–**C**) Volcano plots of differentially expressed gens (DEGs) between PTC and normal tissues in GSE29265, GSE33630, and GSE60542. (**D**) Venn diagram showing the dysregulated anoikis-related genes common to the four datasets. (**E**) Bar graph showing the GO and KEGG analysis. (**F**) PPI network showing the distribution and relationship of the different enriched functions.

The data of PTC was obtained from three GEO cohorts and TCGA database, and the details are shown in Table 1. By comparing tumor tissues and adjacent non-neoplastic tissues, 561 DEGs were obtained from GEO: GSE29265, with 288 genes showing upregulation and 273 showing downregulation in tumor tissues (Figure 2A). 806 DEGs were obtained from GEO: GSE33630, with 459 genes showing upregulation and 347 showing downregulation in tumor tissues (Figure 2B). 745 DEGs were obtained from GEO: GSE60542, with 381 genes showing upregulation and 364 showing downregulation in tumor tissues (Figure 2C). The DEGs obtained from the GEO datasets were intersected with the 551 anoikis-related genes to obtain ADGs. 64 ADGs overlapped between the four datasets (Figure 2D). The modulation-specific biological processes and pathways features of the 64 ADGs were then analyzed. Results were visualized by bar graphs and PPI networks. The GO and KEGG analyses showed that enrichments were mainly focused on positive regulation of cell migration, pathways in cancer, response to wounding, proteoglycans in cancer, and positive regulation of programmed cell death et al. (Figure 2E, 2F).

**Table 1:** Overview of each dataset associated with PTC from GEO and TCGA.

	**Platform**	**Cases of normal**	**Cases of tumor**	**Scanned items**	**Clinical files**
GSE29265	GPL570	20	20	mRNA	No
GSE33630	GPL570	45	49	mRNA	No
GSE60542	GPL570	30	33	mRNA	No
TCGA	NA	−	498	mRNA	Yes

### Construction of the ADGs-related prognostic model

**Figure 3:**
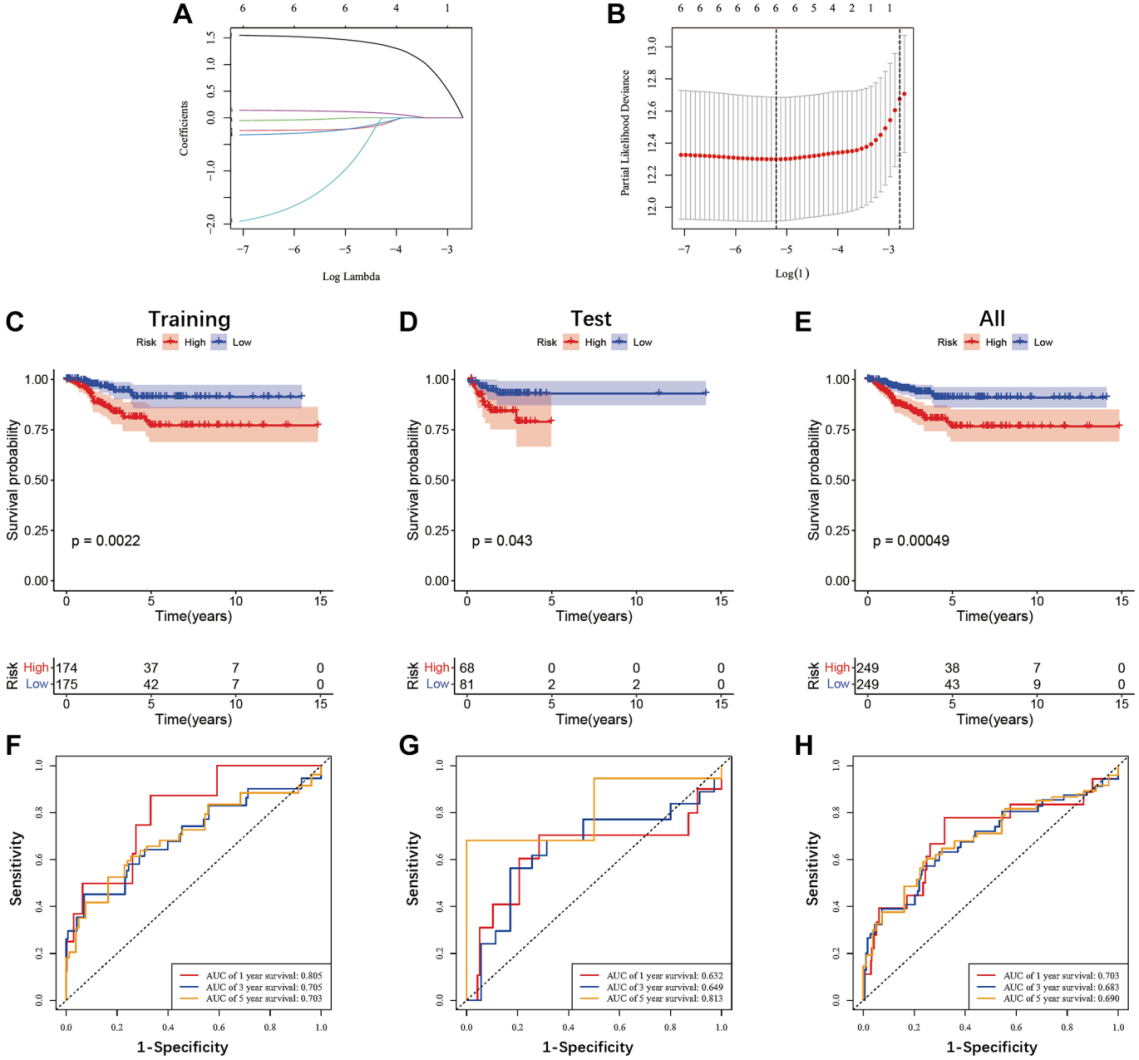
**Prognostic analysis of the six anoikis-related genes signature in the TCGA cohort.** (**A**, **B**) LASSO regression was performed with the minimum criteria. (**C**–**E**) Kaplan-Meier survival analyses comparing the progress-free survival of patients in the high- and low-risk groups were conducted in both the TCGA training cohort (*P* = 0.0022), test cohort (*P* = 0.043), and overall cohort (*P* = 0.00049). (**F**–**H**) The ROC curves validated the prognostic performance of the risk score in both the TCGA training, test, and overall cohort.

Gene expression data (FPKM) with corresponding patient information were obtained from TCGA database of 498 PTC patients. The clinical characteristics of these two cohorts are summarized in Table 2, and there are no significant clinical differences between these two cohorts. Univariate Cox regression analysis in training cohort showed that six ADGs were significantly associated with tumor progression (Supplementary Table 1). Both six genes were subsequently identified as prognostic signature in a LASSO Cox regression analysis (Figure 3A, 3B). Thus, the anoikis-related genes signature was established based on the coefficient in the LASSO regression analysis. A risk score for each patient was calculated as follows: 1.4829 × (expression of EZH2) + (−0.2218) × (expression of PRKCQ) + (−0.0188) × (expression of CD36) + (−0.2417) × (expression of INHBB) + (−1.1662) × (expression of TDGF1) + (0.1198) × (expression of MMP9).

**Table 2:** The clinical characteristics of 498 PTC patients in TCGA.

**Categories**	**Total (*n* = 498)**	**Training (*n* = 349)**	**Test (*n* = 149)**	***P*-value**
**Age at diagnosis**				
<55	333 (66.9)	239 (68.5)	94 (63.1)	0.242
≥55	165 (33.1)	110 (31.5)	55 (36.9)	
**Gender**				
Male	134 (26.6)	86 (24.6)	48 (32.2)	0.081
Female	364 (73.1)	263 (75.4)	101 (67.8)	
**T stage**				
T1	143 (28.7)	97 (27.8)	46 (30.9)	0.174
T2	162 (32.5)	118 (33.8)	44 (29.5)	
T3	168 (33.7)	112 (32.1)	56 (37.6)	
T4	23 (4.6)	20 (5.7)	3 (2.0)	
Tx	2 (0.4)	2 (0.6)	0 (0.0)	
**Pathological stage**				
Stage I	281 (56.4)	208 (59.6)	73 (49.0)	0.051
Stage II	51 (10.2)	34 (9.7)	17 (11.4)	
Stage III	110 (22.1)	66 (18.9)	44 (29.5)	
Stage IV	54 (10.8)	39 (11.2)	15 (10.1)	
Unknown	2 (0.4)	2 (0.6)	0 (0.0)	
**Status**				
Progress free	448 (90.0)	314 (90.0)	134 (89.9)	0.990
Progress	50 (10.0)	35 (10.0)	15 (10.1)	

### Estimations of the prognosis signature in the TCGA database

**Figure 4:**
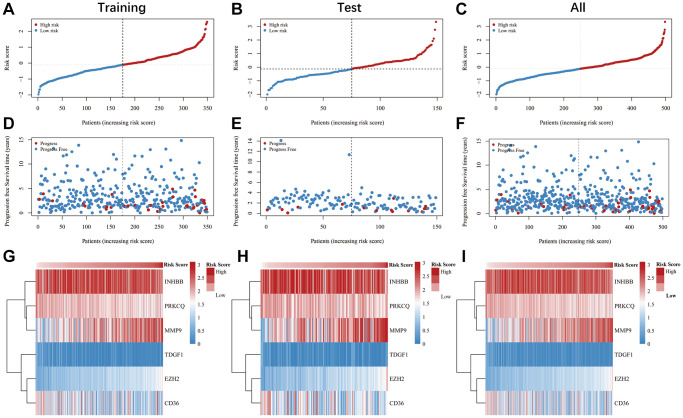
**Verification of the anoikis-related genes signature was conducted in the training, test, and overall cohorts.** (**A**–**F**) Dot plots illustrating the survival and risk score for the training, test, and overall cohorts. (**G**–**I**) The heatmaps of six anoikis-related genes in the training, test, and overall cohorts.

**Figure 5:**
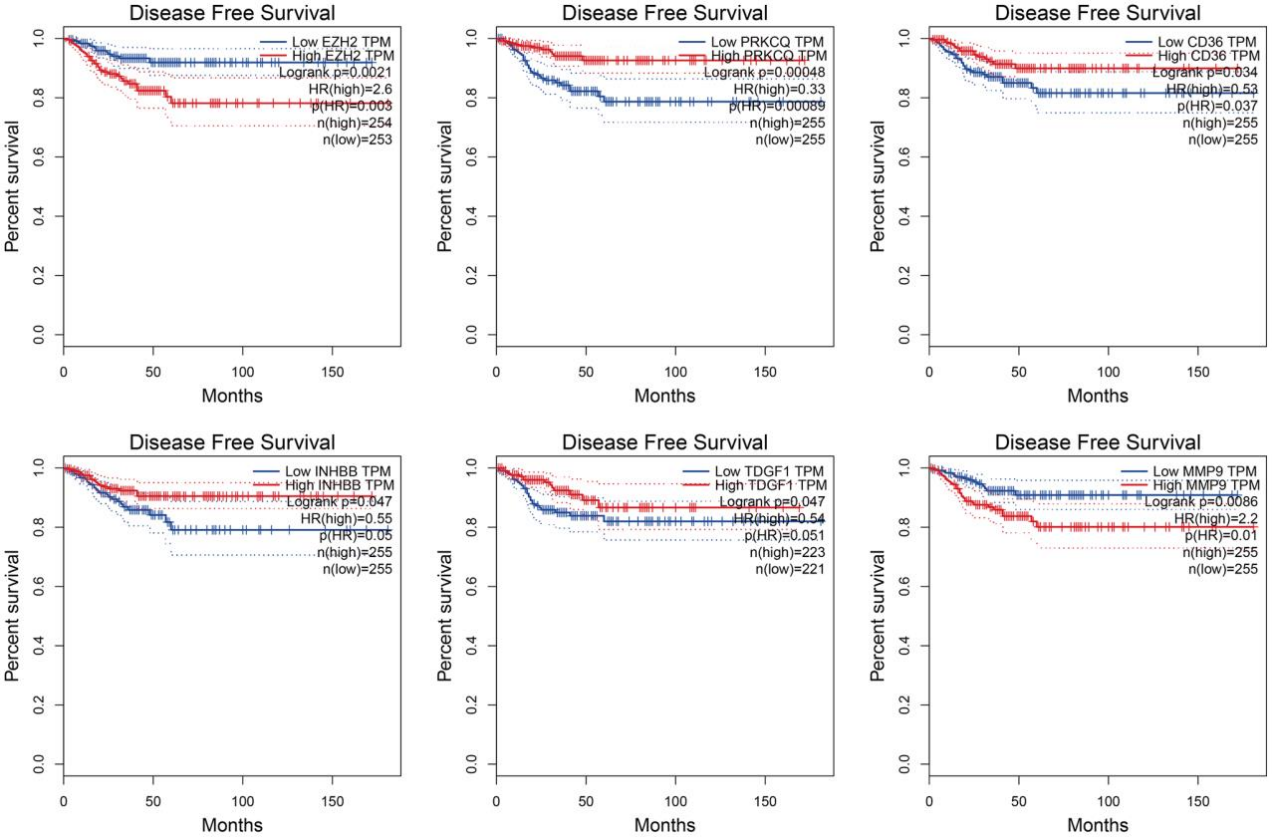
Kaplan-Meier curves of genes associated with the six gene prognostic risk signature.

We employed K-M and ROC curves to evaluate the prognostic significance of the anoikis-related prognostic signature across training, test, and overall cohorts. The K-M analysis showed that patients with high-risk scores had significantly worse progression-free survival (FPS) rates compared to patients with low-risk score in both the training (*P* = 0.0022), test (*P* = 0.043), and overall (*P* = 0.00049) cohorts (Figure 3C–3E). The AUC values for 1-, 3-, and 5-year FPS rates obtained from the prognostic signature in the training cohort were 0.805, 0.705, and 0.703, respectively (Figure 3F), while those in the test cohort were 0.632, 0.649, and 0.813, respectively (Figure 3G), and in the overall cohort were 0.703, 0.683, and 0.69, respectively (Figure 3H). The distribution of the risk score is shown in Figure 4. The progression rate of PTC patients increases proportionally with their risk scores (Figure 4A, 4D). Furthermore, this pattern remains consistent across both the test (Figure 4B, 4E) and overall (Figure 4C, 4F) cohorts. Significantly, patients with high-risk scores exhibited elevated expression levels of EZH2 and MMP9, while PRKCQ, CD36, INHBB, and TDGF1 demonstrated decreased expression levels across all three cohorts (Figure 4G–4I). Utilizing the GEPIA database, we conducted survival analysis on the six genes featured in the prognostic signature. Our findings revealed that PRKCQ, CD36, INHBB, and TDGF1 were correlated with better prognosis and disease-free survival (DFS) in THCA patients, whereas EZH2 and MMP9 were linked to an unfavorable prognosis and reduced DFS among individuals with THCA (Figure 5).

### Establishment and evaluation of the nomogram

**Figure 6:**
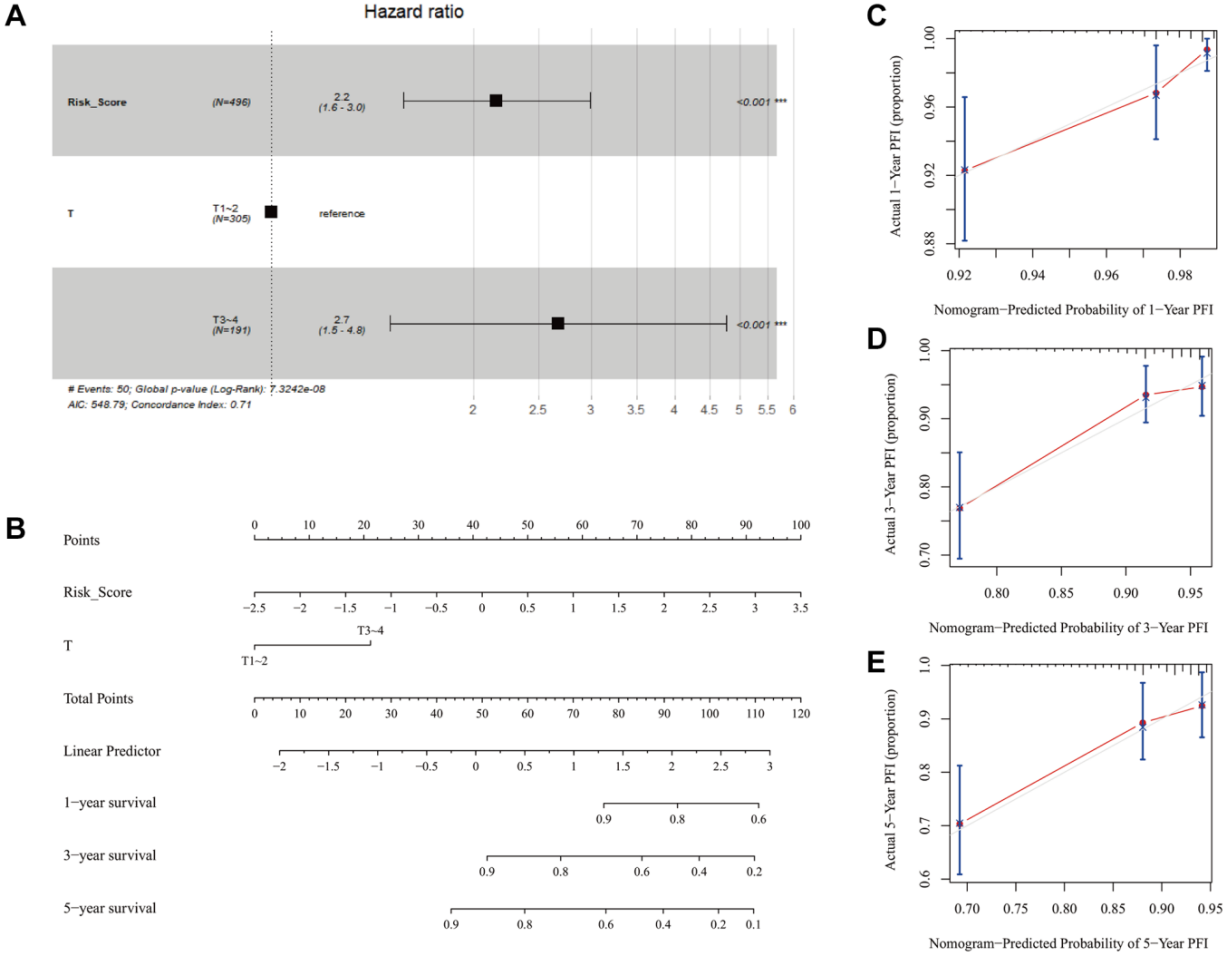
**Forest plot of the multivariate Cox regression analysis and the construction and evaluation of the nomogram.** (**A**) Forest plot showing the risk score and T stage were the significant prognostic factors in predicting progress-free survival in patients with PTC. (**B**) Nomogram based on the risk score of the model and clinical information of PTC patients in the TCGA cohort. (**C**–**E**) Calibration curves of the nomogram for the probability of 1-, 3- and 5-years.

The univariate and multivariate Cox regression analyses showed that the risk score and T stage were the independent significant prognostic factors in predicting PFS in patients with PTC (Supplementary Table 2 and Figure 6A). We subsequently constructed a predictive nomogram with these factors (Figure 6B). Besides, the C-index calculated by R for the nomogram was 0.712, suggesting that the nomogram had a superior predictive performance. The calibration curves were used to evaluate the accuracy of the nomogram, in which a standard curve represented the best prediction. The predicted outcomes of 1-, 3- and 5-year progress-free survival rates showed excellent consistency (Figure 6C–6E).

### Functional enrichment analysis of the DEGs between the high- and low-risk patients

**Figure 7:**
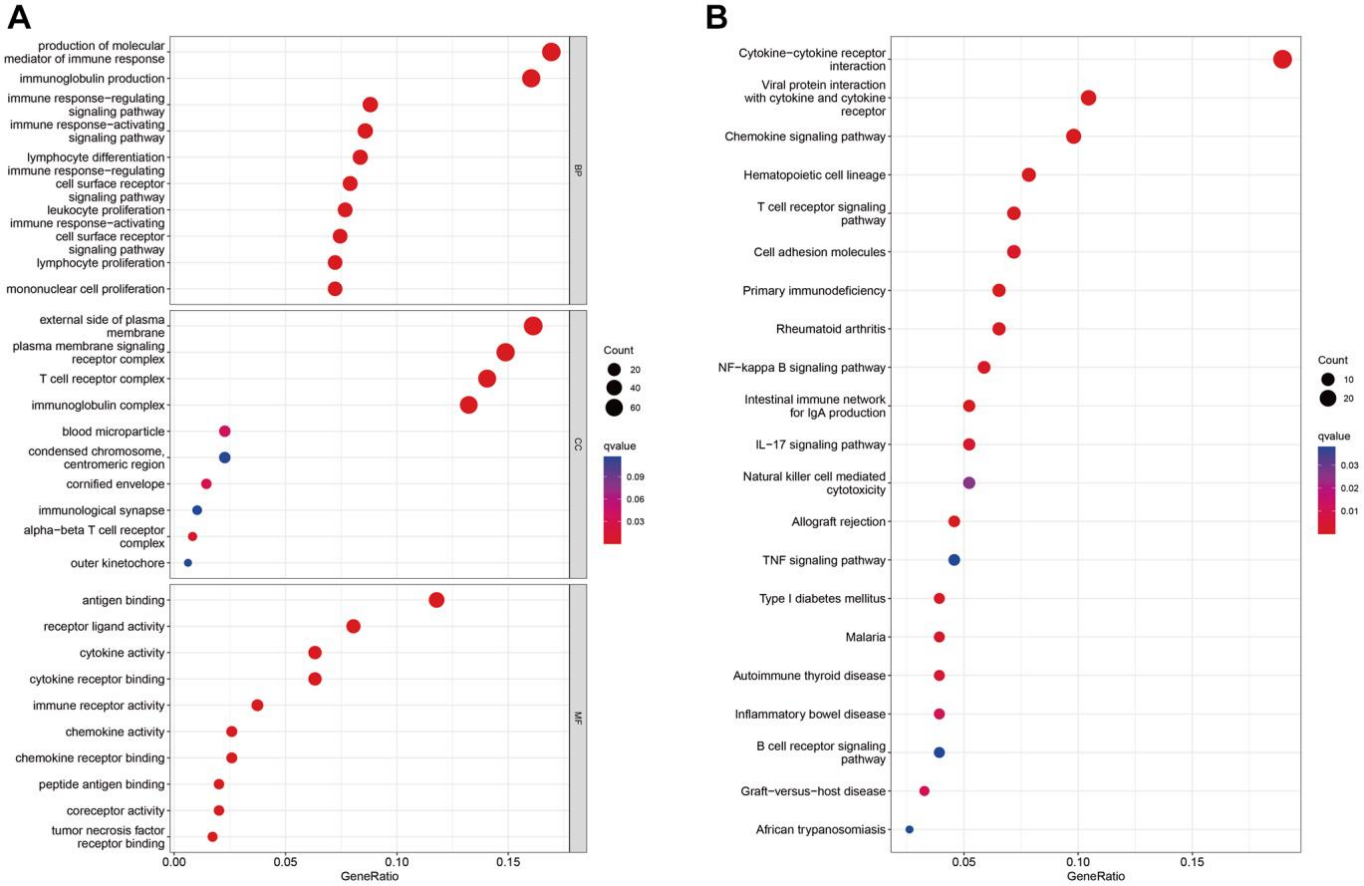
**Enrichment analysis of differentially expressed genes between patients in high- and low-risk groups in the TCGA cohort.** (**A**, **B**) GO (**A**) and KEGG (**B**) analysis results of differentially expressed genes.

567 DEGs were obtained between high- and low-risk groups (Supplementary Figure 1). In order to gain a deeper comprehension of the potential biological mechanisms underlying the DEGs associated with high- and low-risk patients, we performed an analysis utilizing the GO and KEGG databases. The investigation revealed that the biological processes primarily encompassed the production of molecular mediator of immune response, immunoglobulin production, and immune response-regulating and activating cell surface receptor signaling pathway. Additionally, the molecular functions were found to be associated with antigen binding, receptor ligand activity, cytokine activity, and cytokine receptor binding. The cellular components analyzed in this study encompassed the external side of plasma membrane, plasma membrane signaling receptor complex, T cell receptor complex, and immunoglobulin complex. (Figure 7A, Supplementary Table 3). Through KEGG analysis, it was determined that these DEGs were primarily associated with cytokine-cytokine receptor interaction, viral protein interaction with cytokine and cytokine receptor, chemokine signaling pathway, and hematopoietic cell lineage (Figure 7B, Supplementary Table 4).

### Assessment of the immune microenvironment in PTC

**Figure 8:**
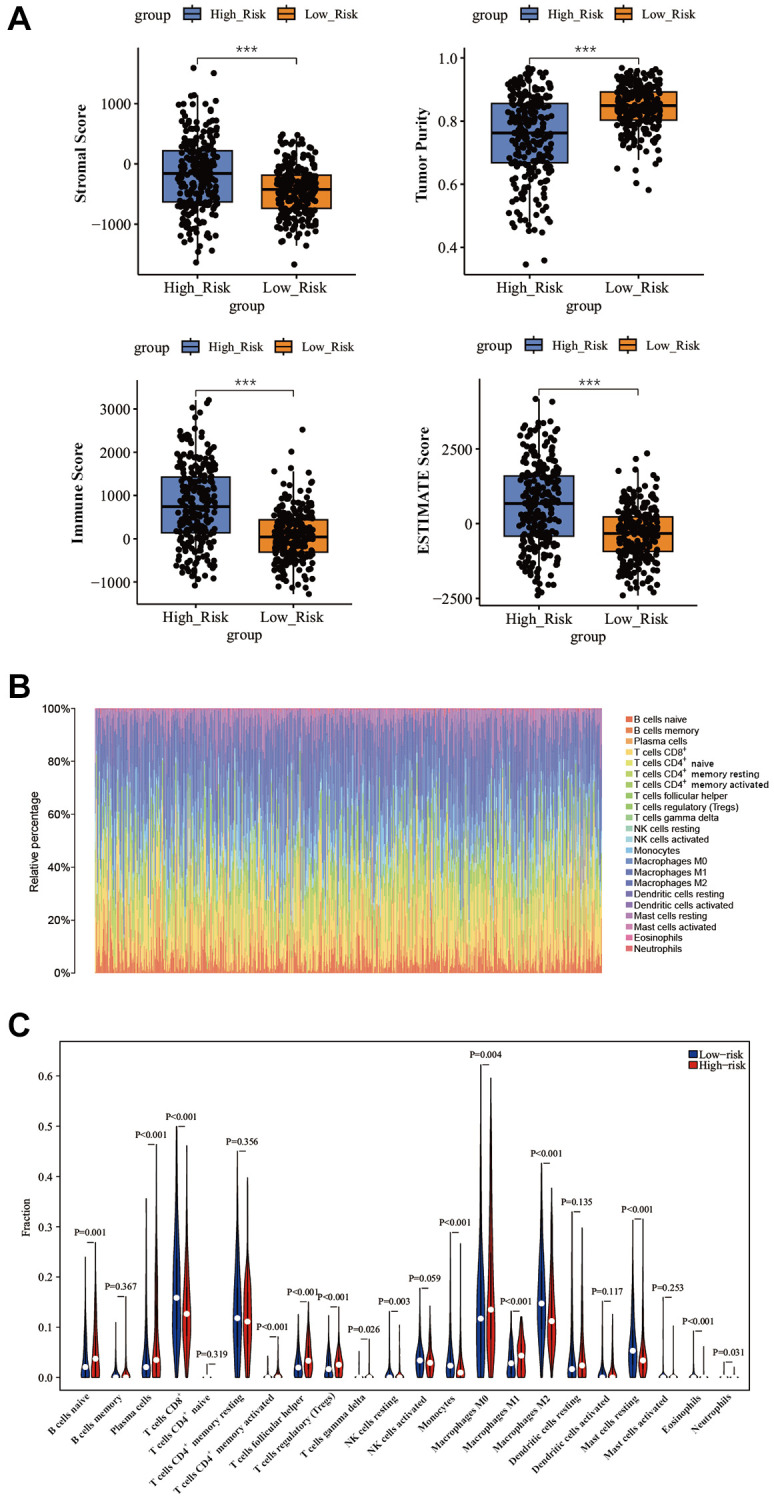
**Correlation between immune cell infiltration and different risk scores.** (**A**) Differences of TME between different risk groups by ESTIMATE algorithm. (**B**) Immune cell proportions for each tumor patient. (**C**) Analyzing the immune cell infiltration levels of PTC samples between different risk groups by CIBERSORT algorithm. ^*^P < 0.05; ^**^P < 0.01; ^***^P < 0.001.

The ESTIMATE algorithm was used to calculate the stromal score, tumor purity, immune score, and ESTIMATE score. The high-risk group had a higher stromal score, immune score, and ESTIMATE score, and a lower tumor purity than the low-risk group (Figure 8A). To explore the correlation between anoikis-related genes signature and immune landscape in PTC, we employed the CIBERSORT algorithm to assess the relative proportions of 22 immune cell types in a cohort of 498 PTC patients from the TCGA database (Figure 8B). Immune cells, including B cells naïve, plasma cells, T cells CD8^+^, T cells CD4^+^ memory activated, T cells follicular helper, T cells regulatory (Tregs), T cells gamma delta, NK cells resting, monocytes, macrophages M0, macrophages M1, macrophages M2, mast cells resting, eosinophils, and neutrophils were statistically different between the high- and low-risk groups (Figure 8C).

### Prediction of drug sensitivity and immunotherapy efficacy

**Figure 9:**
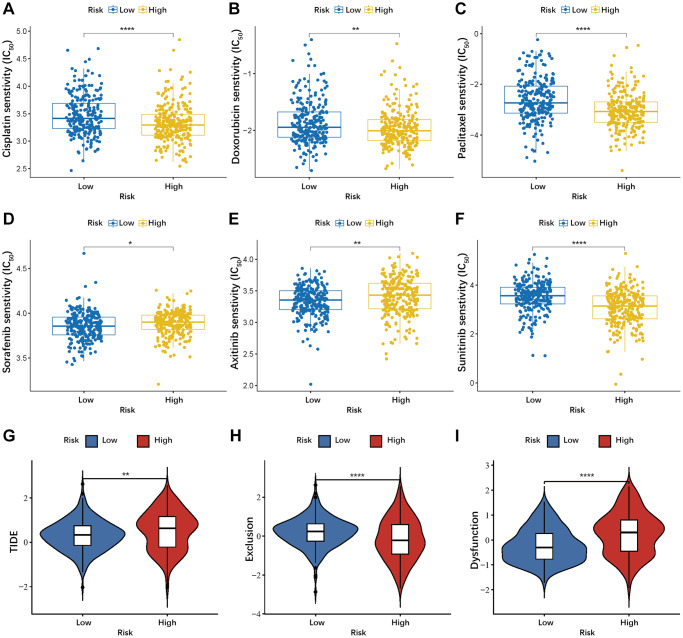
**Evaluation the value of the anoikis-related prognostic model in drug sensitivity and immunotherapy.** (**A**–**F**) Correlation between risk score and drug sensitivity. (**G**–**I**) Differences in TIDE, Exclusion, and Dysfunction between low- and high-risk groups. ^*^P < 0.05; ^**^P < 0.01; ^***^P < 0.001; ^****^P < 0.0001.

Using the R package “pRRophetic”, we investigated the IC_50_ values of six common chemotherapeutic and targeted drugs (Cisplatin, Doxorubicin, Paclitaxel, Sorafenib, Axitinib, and Sunitinib) in both the high- and low-risk groups of PTC patients. PTC patients in the low-risk group demonstrated greater sensitivity to Sorafenib and Axitinib. Conversely, PTC patients in the high-risk group exhibited increased sensitivity to Cisplatin, Doxorubicin, Paclitaxel, and Sunitinib (Figure 9A–9F). To evaluate the risk model’s value in immunotherapy, we compared the TIDE and other immune-related scores between high- and low-risk groups. The high-risk group showed higher TIDE score and Dysfunction score, and lower Exclusion score (Figure 9G–9I), indicating a limited immunotherapy benefit for PTC patients in the high-risk group.

## DISCUSSION

An important aspect of cancer progression is the ability of tumor cells to detach from their primary site and invade surrounding tissues or spread to distant organs [25, 26]. In PTC, Lymph node metastasis has been associated with a lower overall survival rate [27], furthermore, distant metastasis serves as the primary cause of death in PTC patients [28]. Therefore, construction of a metastasis-related signature to predict the prognosis may provide important insights into disease management and early intervention [29]. Anoikis, a form of programmed cell death, is initiated when cells lose adhesion to the extracellular matrix [30]. A pivotal process in metastasis involves the capability of cancer cells to survive without adhesion, evading anchorage-dependent cell death [31].

Under normal physiological conditions, anoikis serves as a safeguard, thwarting the survival of detached cells and the formation of secondary tumors [32]. Nonetheless, cancer cells can evolve strategies to elude anoikis, thus bolstering their metastatic potential [33]. Anoikis resistance in PTC arises from intricate pathways, underscoring the significance of targeting anoikis-related genes to counteract PTC progression and metastasis. Multifaceted analyses reveal the intricate interplay among diverse factors influencing anoikis resistance in PTC [34–38]. Understanding the molecular mechanisms underlying anoikis resistance in PTC is crucial for developing targeted therapies to prevent metastasis and improve patient outcomes [39].

This study was the first attempt at establishing an anoikis-related prognostic genes signature in PTC. In our study, we analyzed DEGs in PTC patients from three GEO datasets. 64 ADGs were subsequently selected by intersecting DEGs and anoikis-related genes. Functional analysis showed positive regulation of cell migration, pathways in cancer, response to wounding, and et al were major enriched, which highlighted the role of ADGs in tumor progression. By using univariate and LASSO Cox regression analyses, we proposed 6-gene signature, including EZH2, PRKCQ, CD36, INHBB, TDGF1, and MMP9. Their role in the process of anoikis resistance has been demonstrated.

EZH2 could reduce ITGα2 transcription, leading to decreased focal adhesions between the extracellular matrix and the cytoskeleton, enhancing cell mobility and increase anoikis resistance [40]. PRKCQ could promote anoikis resistance via kinase-activity-dependent stimulation of Erk/MAPK in breast cancer [41]. Blocking CD36 function or reducing its transcription could limit demethyl fruticulin A intake and integrin sequestration, restoring cell division and preventing anoikis [42]. INHBB could suppress anoikis resistance and migration of nasopharyngeal carcinoma cells by the TGF-β signaling pathway [43]. TDGF1 could enhance the anoikis resistance and the invasion ability of breast cancer cells [44]. MMP9 exerts its effects on the epithelium by cleaving one or more components of cell-cell junctions and triggering anoikis [45].

The risk score was subsequently calculated, and the ROC curves and AUC values confirmed the predictive power of our signature. In addition, our K-M analyses showed that the expression of each of these six genes was associated with the prognosis of PTC. Furthermore, we developed a nomogram incorporating the risk score and T stage. The nomogram demonstrated excellent predictive ability, as indicated by a high C-index of 0.712. This may have the potential to introduce novel insights into clinical decision-making processes. Additionally, we divided patients into high- and low-risk groups by the median risk score, and performed enrichment analysis, immune infiltration analysis, and drug sensitivity evaluation between two groups of PTC patients in TCGA.

The GO enrichment analysis showed that the biological processes involved the production of molecular mediator of immune response, immunoglobulin production, and immune response signaling pathway, etc., which indicated that our prognostic model may be closely related to the biological immune process. Thus, these results motivated us to study the relationship between our prognostic model and tumor immune microenvironment (TME). Additionally, the KEGG enrichment analysis results showed the significance of cytokine-cytokine receptor interaction, which could regulate the immune mechanism of PTC patients and then affect the progression, metastasis and migration of the disease [46]. Besides, the KEGG enrichment analysis results highlighted the significance of pathways such as chemokine signaling pathway. The chemokine could be involved in recruitment and maintenance of immune cells within the THCA microenvironment [47], and this process may involve thyroid cancer cell growth, aggressiveness and metastasis [48].

The immune microenvironment refers to the complex network of immune cells, cytokines, chemokines, and other factors present within the tumor microenvironment [49]. A balanced TME can recognize and eliminate cancer cells, a process known as immunosurveillance [50]. However, tumors often develop various strategies to evade immune recognition and destruction, leading to immune evasion and tumor growth [51]. Therefore, we explored the TME between our two groups. The high-risk group exhibited higher stromal score, immune scores, and ESTIMATE score, and lower tumor purity compared to the low-risk group. Studies have suggested that an increase in stromal cells is associated with a poorer prognosis in THCA, a finding consistent with our results [52]. Furthermore, the augmentation of stromal cells can enhance the expression of diverse immune checkpoints, facilitating tumor cell immune evasion [52].

The differences in immune scores suggested different infiltration of immune cells between the two groups. We found that the infiltration of immune cells, including B cells naïve, plasma cells, T cells CD4^+^ memory activated, T cells follicular helper, T cells regulatory (Tregs), T cells gamma delta, macrophages M0, and macrophages M1, were higher in high-risk group, while T cells CD8^+^, NK cells resting, monocytes, macrophages M2, mast cells resting, eosinophils, and neutrophils were lower in high-risk group. These findings showed that aberrant immune cell infiltration may facilitate the progression of PTC, and may provide guidance for us to further analyze the correlation between TME and anoikis-related genes in PTC.

In this study, we analyzed the drug sensitivity of chemotherapy drugs and targeted drugs commonly used for PTC patients. The high-risk group showed increased sensitivity to Sorafenib and Axitinib, but reduced sensitivity to Cisplatin, Doxorubicin, Paclitaxel, and Sunitinib. Furthermore, tumor immunotherapy has gained significant popularity, but the immunotherapy of THCA is still in the initial stage of exploration [53]. TIDE scores serve as a predictive tool for patient response to immunotherapy, as they predict the tumor’s potential capacity for immune evasion [24]. In our study, the high-risk group exhibited higher TIDE scores and Dysfunction score, and lower Exclusion score. These findings indicate that PTC patients in the high-risk group may experience high immune escape potential due to immune cell dysfunction, and the immunotherapy efficacy may be limited.

However, our study has certain limitations. Firstly, our research primarily relies on data from public databases, necessitating the need for further cohort studies with larger sample sizes to validate our findings. Secondly, the underlying mechanisms of the association between ARGs and PTC warrant further experimental verification.

## CONCLUSION

In conclusion, we developed a six anoikis-related genes signature capable of predicting progression in PTC patients. Further investigations into the molecular mechanisms underlying these features will enhance our understanding of their impact on PTC progression, potentially providing valuable insights for precision medicine approaches.

## Supplementary Materials

Supplementary Figure 1

Supplementary Tables
