# Supplementary material for: Identification and validation of an anoikis-related genes signature for prognostic implication in papillary thyroid cancer
Source: Aging (Albany NY). 2024 Apr 24;16(8):7405–25. doi: 10.18632/aging.205766 (PMC11087102; doi:10.18632/aging.205766)
Supplement: Supplementary Figure 1 [file aging-16-205766-s001.pdf]

## SUPPLEMENTARY FIGURE

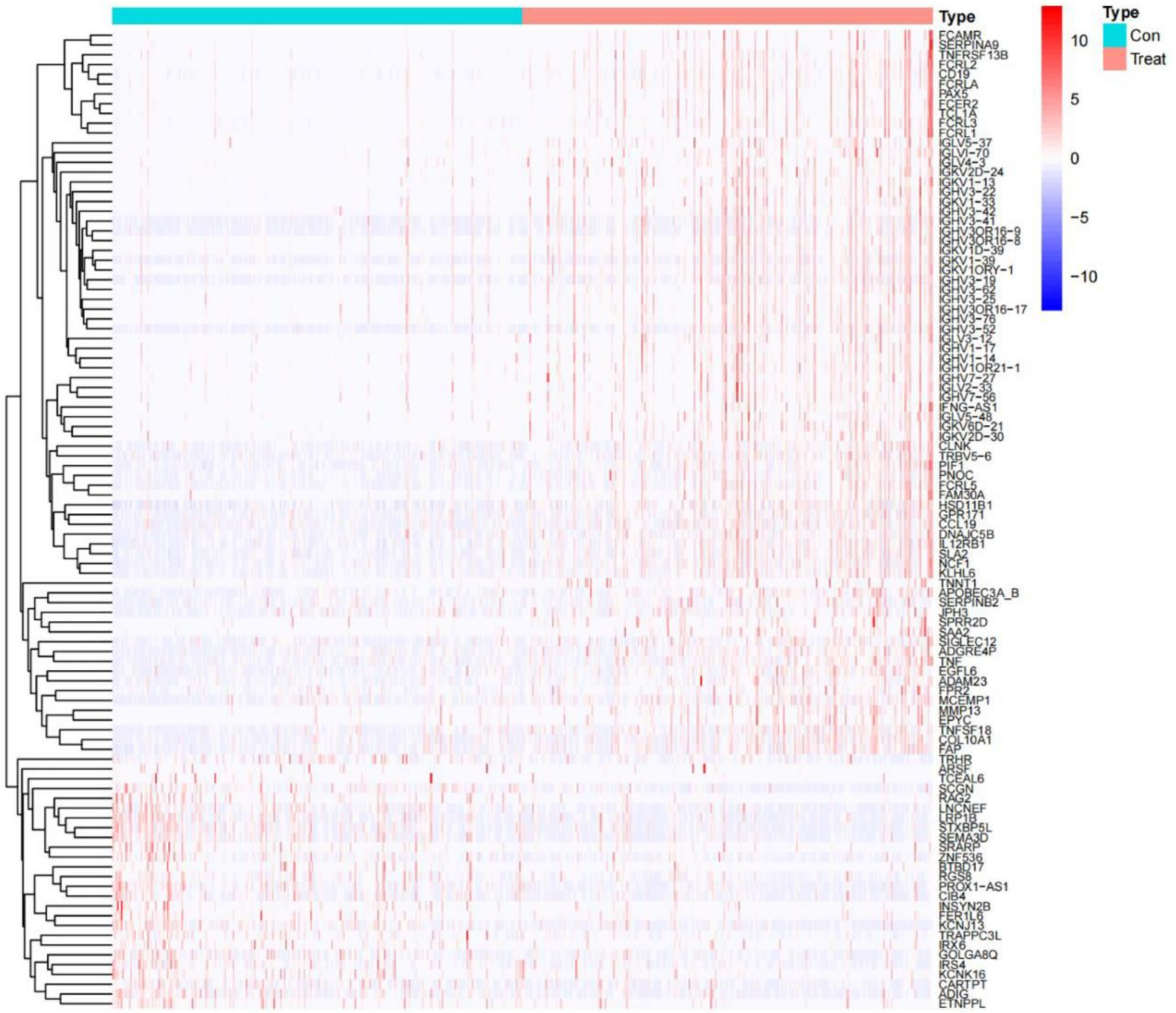


**Supplementary Figure 1. Heatmap of the DEGs between high- and low-risk groups.** Abbreviations: Con: low-risk group; Treat: high-risk group.
