# Supplementary material for: Identification and validation of an anoikis-related genes signature for prognostic implication in papillary thyroid cancer
Source: Aging (Albany NY). 2024 Apr 24;16(8):7405–25. doi: 10.18632/aging.205766 (PMC11087102; doi:10.18632/aging.205766)
Supplement: Supplementary Tables [file aging-16-205766-s002.pdf]

SUPPLEMENTARY TABLES

Supplementary Table 1. The results of univariate Cox regression analysis of the 64 ARGs.

Gene	HR	HR.95L	HR.95H	*P*-value
PDK4	0.813384	0.418117	1.582319	0.542944
CXCL12	0.672002	0.341532	1.322236	0.249698
TNFRSF10B	1.618241	0.815094	3.212761	0.16893
F10	0.678121	0.344754	1.333844	0.260432
PRKCQ	0.371878	0.174224	0.793766	0.010558*
BLNK	1.730454	0.871564	3.435744	0.117085
CEACAM6	1.135419	0.583849	2.208067	0.708219
FN1	1.430055	0.727176	2.812329	0.299886
ITGA2	1.289573	0.660118	2.519243	0.456675
TIMP1	0.936598	0.482296	1.818835	0.846622
CCND1	0.700209	0.359639	1.363293	0.294483
PLAUR	1.334809	0.678455	2.626137	0.402929
LGALS3	0.994759	0.512557	1.930607	0.987608
SFN	1.436135	0.73027	2.824274	0.294188
CLDN1	1.160057	0.596391	2.256459	0.661843
CDKN1A	0.784467	0.403266	1.52601	0.474594
PLAU	1.003439	0.517052	1.947368	0.991903
BID	0.900536	0.463154	1.750962	0.757468
FAS	0.817365	0.420266	1.589671	0.552373
LAMB3	1.248822	0.639242	2.439696	0.515482
CDH2	1.022473	0.526885	1.984208	0.947618
SERPINA1	1.080092	0.555243	2.101063	0.820467
MET	1.116252	0.573934	2.171015	0.745915
INHBB	0.435113	0.215994	0.876521	0.01987*
H19	NA	NA	NA	NA
CRYAB	0.605292	0.30488	1.201714	0.151339
IL1RAP	1.028818	0.529971	1.997215	0.933101
LMO3	0.56777	0.285925	1.12744	0.105824
CDH3	0.95003	0.489177	1.845052	0.879689
TGFBR3	0.609496	0.30695	1.210246	0.15715
COL13A1	0.840768	0.43232	1.63511	0.609305
TNFRSF12A	0.722052	0.370535	1.407043	0.338706
TIAM1	1.151504	0.588941	2.251433	0.680073
PRDM1	1.238724	0.636916	2.409165	0.528186
S100A11	1.449379	0.736937	2.850583	0.28218
SLPI	0.856398	0.441236	1.662191	0.646836
CXCL14	0.677088	0.346172	1.324339	0.254593
LTF	0.516769	0.252919	1.055873	0.070167
CD36	0.41095	0.197321	0.855865	0.017512*
PAK3	0.769079	0.395404	1.495895	0.439216
TDGF1	0.430439	0.206679	0.896448	0.024322*
PPARG	0.828337	0.425794	1.611445	0.579105
CD151	1.047616	0.537916	2.040281	0.891207
CRABP2	1.390912	0.706797	2.737189	0.339427

DAPK2	0.871217	0.44879	1.691257	0.683753
ITGA3	1.368697	0.6958	2.692343	0.36322
MUC1	1.376781	0.700026	2.707791	0.354168
S100A4	1.123145	0.577457	2.184502	0.732238
ETV4	0.630625	0.320552	1.240635	0.181742
NOX4	0.952772	0.490779	1.849662	0.88634
KL	1.123588	0.577429	2.186328	0.731537
SPP1	1.311797	0.671443	2.562857	0.427049
TLE1	0.921292	0.474671	1.788143	0.808558
CXCL8	1.015485	0.521409	1.977739	0.963962
LGALS1	0.764104	0.392676	1.486861	0.428289
MMP9	2.23077	1.092652	4.554361	0.027575*
EZH2	3.5077	1.593143	7.723072	0.00183*
LPAR1	0.827049	0.425263	1.608439	0.575795
IFI27	0.553241	0.278584	1.09868	0.090815
TNC	1.184466	0.606089	2.314774	0.620447
ARHGDIB	0.644484	0.327665	1.267634	0.203074
ID2	0.712185	0.36443	1.391781	0.320758
KRT14	0.912598	0.470287	1.770908	0.786857
EFHD2	0.64788	0.329414	1.274226	0.208485

*$P < 0.05$.

Supplementary Table 2. Univariate and multivariate Cox regression analyses of clinical parameters.

	Univariate Cox regression analysis				Multivariate Cox regression analysis				
	HR	HR 95L	HR 95H	*P*-value	Coef	HR	HR 95L	HR 95H	*P*-value
Risk score	2.145516949	1.588845036	2.897225893	6.32E-07*	0.773546345	2.167439128	1.574175931	2.98428675	2.13E-06*
Gender	1.475298614	0.821308281	2.650047555	0.193179932			–		
T stage	2.812650703	1.578300772	5.012355132	0.000451474*	0.985046675	2.677936873	1.502513612	4.772899118	0.000835459*
Focal type	0.912484255	0.520015815	1.601158066	0.749558197			–		

*$P < 0.05$

Supplementary Table 3. The result of GO enrichment analysis.

Ontology	ID	Description	GeneRatio	BgRatio	*p*-value	*p*.adjust	*q*-value	Count
BP	GO:0002377	Immunoglobulin production	71/443	212/18614	2.86E-62	9.08E-59	8.05E-59	71
BP	GO:0002440	Production of molecular mediator of immune response	75/443	328/18614	3.46E-52	5.49E-49	4.86E-49	75
BP	GO:0002768	Immune response-regulating cell surface receptor signaling pathway	35/443	329/18614	1.25E-13	1.33E-10	1.18E-10	35
BP	GO:0002429	Immune response-activating cell surface receptor signaling pathway	33/443	302/18614	3.03E-13	2.40E-10	2.13E-10	33
BP	GO:0002757	Immune response-activating signaling pathway	38/443	423/18614	2.18E-12	1.23E-09	1.09E-09	38
BP	GO:0046651	Lymphocyte proliferation	32/443	306/18614	2.33E-12	1.23E-09	1.09E-09	32
BP	GO:0070661	Leukocyte proliferation	34/443	348/18614	3.23E-12	1.34E-09	1.19E-09	34

BP	GO:0002764	Immune response-regulating signaling pathway	39/443	450/18614	3.38E-12	1.34E-09	1.19E-09	39
BP	GO:0032943	Mononuclear cell proliferation	32/443	313/18614	4.28E-12	1.51E-09	1.34E-09	32
BP	GO:0030098	Lymphocyte differentiation	37/443	422/18614	8.87E-12	2.81E-09	2.49E-09	37
BP	GO:0002253	Activation of immune response	40/443	495/18614	1.58E-11	4.54E-09	4.03E-09	40
BP	GO:0050863	Regulation of T cell activation	34/443	377/18614	2.97E-11	7.86E-09	6.97E-09	34
BP	GO:1903131	Mononuclear cell differentiation	38/443	474/18614	6.58E-11	1.60E-08	1.42E-08	38
BP	GO:0050851	Antigen receptor-mediated signaling pathway	24/443	201/18614	8.87E-11	2.01E-08	1.78E-08	24
BP	GO:0002449	Lymphocyte mediated immunity	29/443	300/18614	1.72E-10	3.64E-08	3.22E-08	29
CC	GO:0019814	Immunoglobulin complex	64/484	108/19518	3.77E-75	1.11E-72	1.08E-72	64
CC	GO:0042101	T cell receptor complex	68/484	147/19518	7.64E-70	1.12E-67	1.10E-67	68
CC	GO:0098802	Plasma membrane signaling receptor complex	72/484	319/19518	2.97E-48	2.90E-46	2.84E-46	72
CC	GO:0009897	External side of plasma membrane	78/484	426/19518	3.79E-45	2.77E-43	2.72E-43	78
CC	GO:0042105	Alpha-beta T cell receptor complex	4/484	12/19518	0.000157807	0.009247507	0.009069766	4
CC	GO:0001533	Cornified envelope	7/484	59/19518	0.000618179	0.030187746	0.029607525	7
CC	GO:0072562	Blood microparticle	11/484	144/19518	0.000942559	0.039452845	0.038694546	11
MF	GO:0003823	Antigen binding	41/348	118/18369	5.80E-41	2.50E-38	2.28E-38	41
MF	GO:0005125	Cytokine activity	22/348	235/18369	7.94E-10	1.71E-07	1.56E-07	22
MF	GO:0005126	Cytokine receptor binding	22/348	270/18369	1.06E-08	1.52E-06	1.39E-06	22
MF	GO:0008009	Chemokine activity	9/348	49/18369	2.99E-07	2.66E-05	2.42E-05	9
MF	GO:0048018	Receptor ligand activity	28/348	497/18369	3.08E-07	2.66E-05	2.42E-05	28
MF	GO:0140375	Immune receptor activity	13/348	141/18369	2.89E-06	0.000208288	0.000189814	13
MF	GO:0042605	Peptide antigen binding	7/348	39/18369	7.53E-06	0.000464576	0.000423371	7
MF	GO:0042379	Chemokine receptor binding	9/348	74/18369	1.06E-05	0.000572551	0.000521769	9
MF	GO:0005164	Tumor necrosis factor receptor binding	6/348	30/18369	1.79E-05	0.000859425	0.000783199	6
MF	GO:0015026	Coreceptor activity	7/348	48/18369	3.11E-05	0.001222713	0.001114266	7
MF	GO:0032813	Tumor necrosis factor receptor superfamily binding	7/348	48/18369	3.11E-05	0.001222713	0.001114266	7
MF	GO:0004222	Metalloendopeptidase activity	10/348	122/18369	0.000110295	0.00397062	0.00361845	10
MF	GO:0042287	MHC protein binding	6/348	42/18369	0.000130633	0.00416982	0.003799983	6
MF	GO:0001664	G protein-coupled receptor binding	16/348	289/18369	0.000135133	0.00416982	0.003799983	16
MF	GO:0045236	CXCR chemokine receptor binding	4/348	18/18369	0.000313967	0.00904225	0.008240257	4

Supplementary Table 4. The result of KEGG enrichment analysis.

ID	Description	GeneRatio	BgRatio	*p*-value	*p*.adjust	*q*-value	Count
hsa04060	Cytokine-cytokine receptor interaction	29/153	297/8622	3.21E-14	6.92E-12	6.28E-12	29
hsa04061	Viral protein interaction with cytokine and cytokine receptor	16/153	100/8622	1.64E-11	1.77E-09	1.61E-09	16
hsa05340	Primary immunodeficiency	10/153	38/8622	7.12E-10	5.12E-08	4.65E-08	10

hsa04640	Hematopoietic cell lineage	12/153	99/8622	1.55E-07	8.37E-06	7.58E-06	12
hsa04062	Chemokine signaling pathway	15/153	192/8622	1.43E-06	6.16E-05	5.59E-05	15
hsa04672	Intestinal immune network for IgA production	8/153	49/8622	1.99E-06	7.17E-05	6.50E-05	8
hsa05330	Allograft rejection	7/153	38/8622	3.84E-06	0.000118548	0.000107455	7
hsa05323	Rheumatoid arthritis	10/153	93/8622	5.28E-06	0.000142496	0.000129163	10
hsa04660	T cell receptor signaling pathway	11/153	121/8622	9.16E-06	0.000219757	0.000199195	11
hsa04064	NF-kappa B signaling pathway	9/153	104/8622	9.01E-05	0.001946043	0.001763957	9
hsa04940	Type I diabetes mellitus	6/153	43/8622	0.000100335	0.00197022	0.001785872	6
hsa04514	Cell adhesion molecules	11/153	158/8622	0.000109874	0.001977739	0.001792687	11
hsa05144	Malaria	6/153	50/8622	0.000236127	0.003868426	0.003506468	6
hsa04657	IL-17 signaling pathway	8/153	94/8622	0.000250731	0.003868426	0.003506468	8
hsa05320	Autoimmune thyroid disease	6/153	53/8622	0.000326537	0.004702139	0.004262173	6
hsa05332	Graft-versus-host disease	5/153	42/8622	0.000825575	0.011145267	0.010102435	5
hsa05321	Inflammatory bowel disease	6/153	65/8622	0.000986645	0.012536201	0.011363224	6
hsa04650	Natural killer cell mediated cytotoxicity	8/153	132/8622	0.002355524	0.028266288	0.025621489	8
hsa04662	B cell receptor signaling pathway	6/153	84/8622	0.003687196	0.041917599	0.037995484	6
hsa05143	African trypanosomiasis	4/153	37/8622	0.003993356	0.041933578	0.038009969	4
hsa04668	TNF signaling pathway	7/153	114/8622	0.004076876	0.041933578	0.038009969	7
